# Students’ Feedback on the Development of a Competency-Based Pharmacy Education (CBPE) at the University of Tartu, Estonia

**DOI:** 10.3390/pharmacy9010045

**Published:** 2021-02-19

**Authors:** Daisy Volmer, Kristiina Sepp, Ain Raal

**Affiliations:** Institute of Pharmacy, Faculty of Medicine, University of Tartu, 50411 Tartu, Estonia; kristiina.sepp@ut.ee (K.S.); ain.raal@ut.ee (A.R.)

**Keywords:** competency, education, pharmacy, Estonia

## Abstract

Increasing need in society to provide collaborative and patient-centered pharmaceutical care has to be addressed in curriculum development. Principles of competency-based pharmacy education (CBPE) could be seen as one solution to the new professional challenges of pharmacists. At the University of Tartu (UT), the Pharmacy curriculum was updated in 2019 to introduce principles of CBPE. The aim of this study was to gather initial students’ feedback on the development of CBPE at the UT. The survey was conducted in the spring semester of the 2019/2020 academic year to collect feedback about all curricula at the UT. All 1st, 3rd, and 5th year pharmacy students (n = 67) were invited and 70.1% (N = 47) of them also participated in this study in order to evaluate the Pharmacy curriculum. Pharmacy students were more complacent with the content and less with the fixed structure of the Pharmacy curriculum. Students emphasized more theoretical knowledge and less practical and transferable skills of the competencies developed over the studies. Initial student feedback on the development of CBPE in Estonia demonstrated that theoretical knowledge needs to be more integrated with practice throughout the curriculum. In the future, more attention should be paid to the development of transferable skills, including digital skills.

## 1. Introduction

To start with, there is an increasing societal demand for professionals who are able to provide evidence based and patient-centered care [1,2]. Thus, it is important to revisit traditional way of teaching and learning to tailor pharmacy education according to changed public expectations. Principles of competency-based pharmacy education (CBPE) covering balanced professional science-based and patient-oriented knowledge and introduced in several countries, could be seen as one solution to the new professional challenges of pharmacists [1,2,3,4,5,6].

The implementation of CBPE is often complicated as competence is multi-dimensional, dynamic, and changes with time, experience and setting [6,7]. It is important to integrate theoretical knowledge, practical skills, and professional behavior to perform as a competent specialist and use all these components dynamically in a particular professional situation [7]. Development of CBPE has to be organized with the help of existing or newly developed frameworks and in tight collaboration with other stakeholders of pharmacy and healthcare sector, as it is essential to be in line to the practical professional needs of a particular country [6,8]. 

Community pharmacies in Estonia provide traditional and extended services. However, public expectations towards pharmacists go beyond the dispensing and compounding of medicines. Patients with chronic conditions and/or polypharmacy need person-centered health counselling, self-care consultation, and medicines management service. The improvement in vaccination coverage and the tackling of resistance to vaccines have already shown that pharmacists have an added value in society [9,10].

The Institute of Pharmacy at the Faculty of Medicine of the University of Tartu (UT) is the only institution in Estonia providing pharmacy education at the university level [9,11]. The Pharmacy curriculum is a five-year integrated bachelor’s and master’s degree (300 ECTS) following the sectorial profession model and the EU directives on pharmaceutical education at higher education institutions [12]. Pharmacy curriculum provides in-depth theoretical knowledge and considers the needs of the healthcare system as well as the general public. It is designed as traditional medical subject-based and pharmaceutical product-oriented curriculum and is the basis for recognition of professional qualification [9,11]. The curriculum was previously updated in 2007. 

### 1.1. Pharmacy Curriculum Developments

In 2014, the Estonian Quality Agency for Higher and Vocational Education emphasized the need to update Pharmacy curriculum and focus on competency-based developments, e.g., to increase analytical and critical thinking and social skills and to develop professionalism and professional identity of pharmacy students [13].

**Figure 1:**
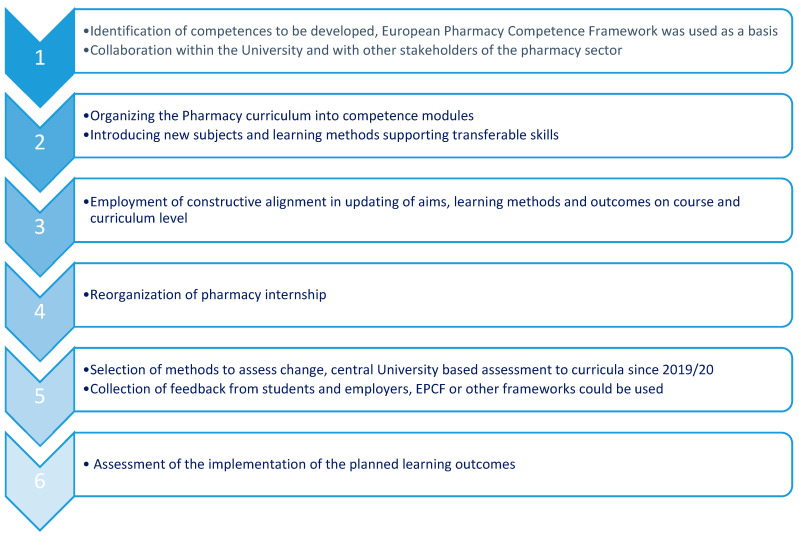
Steps to develop competence-based Pharmacy curriculum at the University of Tartu.

Figure 1 describes the principles of CBPE developments at the UT. The updated curriculum was launched in 2019/20 and the newly educated pharmacists will graduate from the UT in 2024. 

The key points of the new Pharmacy curriculum are as follows:

- integrated bachelor’s and master’s curriculum with a course-based system;
- medical subject-based and pharmaceutical product-oriented design of the curriculum with focus on development of practical clinical and communication skills that are crucial in patient’s education and counselling about medicinal products;
- more efficient linking of theoretical and practical knowledge to support the development of professional competency; compilation of competence-based modules with newly developed outcomes for all subjects in the curriculum, competence-based modules, and Pharmacy curriculum based on the Blooms taxonomy;
- new subjects to increase professional identity and provide comprehensive knowledge about different medicinal products in various settings;
- reorganized pharmacy internship to support more efficient implementation of theoretical knowledge into practice;
- introduction and implementation of contemporary learner-centered teaching methods supported by multiple IT tools [13].

Some of the key-points in the Pharmacy curriculum development are described below in more detail. 

First, the principles of European Pharmacy Competence Framework (EPCF) were used in the curriculum development [14,15]. The framework was employed for evaluation of the Pharmacy curriculum in 2016 by teachers and students as well as by different other stakeholders. An obvious need for a change with an emphasis on patient care competences was seen in the results [11]. 

Second, the Pharmacy curriculum was organized to the competence-based modules as follows:

1. Professional identity and values, scientific thinking (35 ECTS);
2. Practical approach to medicinal products (including pharmacy internship) (49 ECTS);
3. Drug development and manufacture (40 ECTS);
4. Chemical composition and quality of medicinal products (60 ECTS);
5. Patient and medicinal products (55 ECTS);
6. Pharmacotherapy and patient care (27 ECTS) [16].

Third, the principles of constructive alignment [17,18] based on the Bloom’s taxonomy [19] were used to update the course aims, study methods, assessment methods, and learning outcomes. Transferable skills (e.g., teamwork, leadership, personal motivation, time management, listening, verbal and written communication, research and analytical skills, and IT skills) need more attention in the future to increase thinking beyond qualifications and experience. Competence in entrepreneurship was planned to introduce as an obligatory course for opening and managing a community pharmacy. In addition, pharmacy students can take electives in economics and business administration from other faculties at the UT.

Fourth, in collaboration with professional organizations, practicing pharmacists and the University, the structure and content of pharmacy internship were updated as follows:

- themed structure presented in seven modules: Handling of medicinal products at community pharmacy; Counselling of OTC medicines; Counselling of prescription medicines; Compounding of medicines; Counselling of other pharmacy goods (e.g., food supplements and medical devices); Medication Use Review; Hospital pharmacy;
- use of Moodle platform for course materials, communication between supervisors at community pharmacy and students with the Institute;
- use of e-portfolio in the training process;
- regular training of internship supervisors;
- seminars during the internship at the Institute with students and supervisors.

A detailed description of the aims, study, and assessment methods and outcomes produced by the Institute was developed for all modules. Students should develop personal aims and outcomes (could be developed separately for separate modules) in collaboration with supervisor at community/hospital pharmacy. Students’ assignments are prepared by the Institute and internship supervisors. Students post the assignments to e-portfolio with reflection to their professional knowledge and counselling skills. At the end of pharmacy internship, students can collect the e-portfolio and use it during their working career. Students have to present the e-portfolio with personal reflection to aims and outcomes achieved/not achieved during pharmacy internship and self-evaluation of professional knowledge and skills before and after pharmacy internship in order to complete the internship. In April 2019, the OSCE test was first used after a pharmacy internship for the evaluation of clinical and communication skills of the pharmacy students about prescription and OTC medicines [13].

### 1.2. Participation at and Feedback on Curriculum Development

Representatives of professional organizations, Faculty of Medicine, and pharmacy student organization are involved in the development of the Pharmacy curriculum via the Institute Council and Pharmacy Educational Programme Committee. Pharmacy students give feedback to the courses (including teaching quality) via the Study Information System (SIS). Pharmaceutical Society of the UT—a professional organization of pharmacy students provide independent quality assessment of the Pharmacy curriculum in addition to the SIS evaluation. Since the beginning of COVID-19 pandemic in March 2020, the teaching has been crucial as most of it was enabled remotely and, thus, new methods were introduced for student learning and assessment.

The aim of this study was to gather initial students’ feedback on the development of competency-based pharmaceutical education (CBPE) at the University of Tartu.

## 2. Materials and Methods 

### 2.1. Study Design and Sample

At the UT, the curriculum feedback survey was first conducted in the spring semester of the 2019/2020 academic year for all curricula, including Pharmacy curriculum. In integrated studies the feedback was collected from the 1st, 3rd, and 5th year (second, sixth and tenth semester) students. All 1st (n = 23), 3rd (n = 28), and 5th (n = 16) year pharmacy students (n = 67) were invited to participate. The completion of the questionnaire was voluntary.

### 2.2. Study Instrument

The electronic survey instrument that was developed at the Institute of Education, UT, was divided into four sections: curriculum (6 items), learning environment and academic affairs (6 items), development of student competences over the studies (20 items) and support system (7 items). A 4-point Likert scale (4 = I agree, 3 = I somewhat agree, 2 = I somewhat disagree, 1 = I disagree) was used to give replies. If the respondents were unable to rate certain statements, they could select the option “Not applicable”. These answers have been left out from the results. Students were left with a possibility to give additional comments in all sections, if needed.

### 2.3. Data Analysis

Results were calculated if at least five students gave feedback for the respective section separately for all three study years. The results are presented as the average with SD (Appendix A) of all responses separately for each statement given by pharmacy students and compared with replies by separate study year. In addition, the average results were compared on three different levels: Pharmacy curriculum, Faculty of Medicine (medicine, dentistry, pharmacy, sport sciences and physiotherapy) and UT (Appendix B). 

## 3. Results

### 3.1. Respondents

A total of 70.1% (n = 47) of selected study sample (n = 67) participated in the survey: first year 34.1% (n = 16), third year 40.4% (n = 19), and fifth year 25.5% (n = 12).

### 3.2. Pharmacy Curriculum

**Figure 2:**
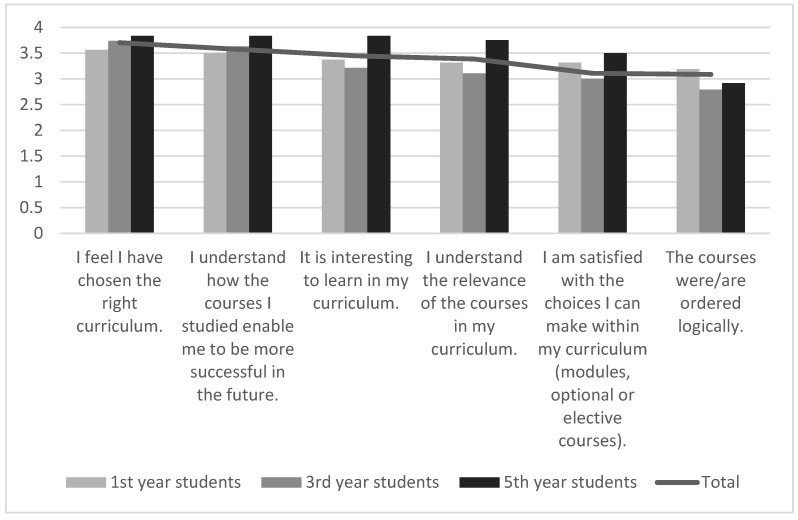
Ratings for the Pharmacy curriculum, average results: total, 1st, 3rd, and 5th year pharmacy students.

Pharmacy students were more satisfied with the selected specialty and the content of the curriculum than with its organization. They wanted more possibilities for flexible selection of obligatory and/or elective subjects. It was also noted (more by third and fifth year students, whose studies are based on the previous curriculum) that the placement of the subjects in the curriculum could be more logical. For all statements, except for the placement of subjects in the curriculum, the results obtained from 5th year students were higher than the results given by first and third year students (Figure 2).

### 3.3. Learning Environment and Academic Affairs

**Figure 3:**
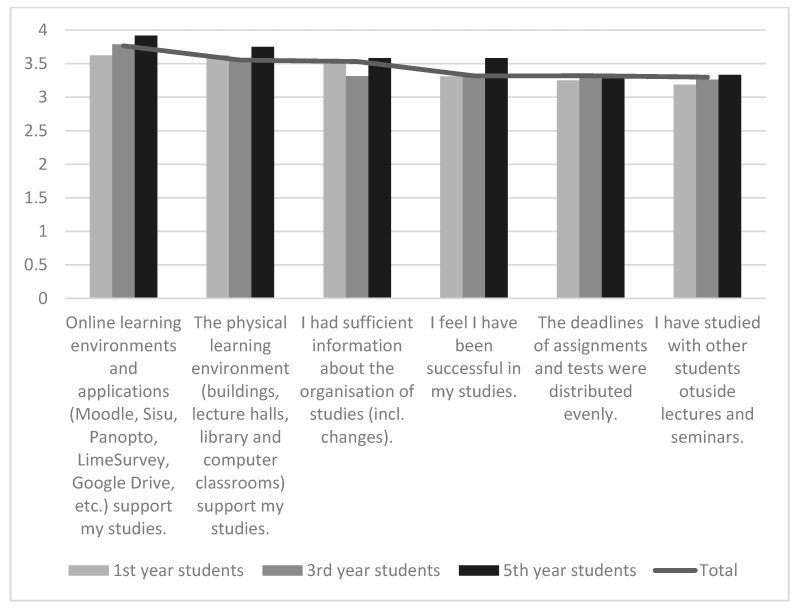
Ratings to learning environment and academic affairs, average results: total, 1st, 3rd, and 5th year pharmacy students.

Many students agreed that web-based environments and applications support their learning. The opinion of students from all three academic years coincided in terms of studying together outside of the classroom. This study method could have been used more at all study levels. Compared to other years, fifth year students were more convinced that they would be successful in their studies (Figure 3). 

### 3.4. Development of Competencies over the Studies

Among all pharmacy students, acquisition of theoretical knowledge and corresponding research methods were mentioned as most important competencies. Students agreed that existing theoretical knowledge can be used in solving practical problems. Students also highlighted the ability to evaluate and analyze professional information critically. In the future, it is important to introduce study principles of university to students as some of them were not able to plan their studies even being already on the final year. 

**Figure 4:**
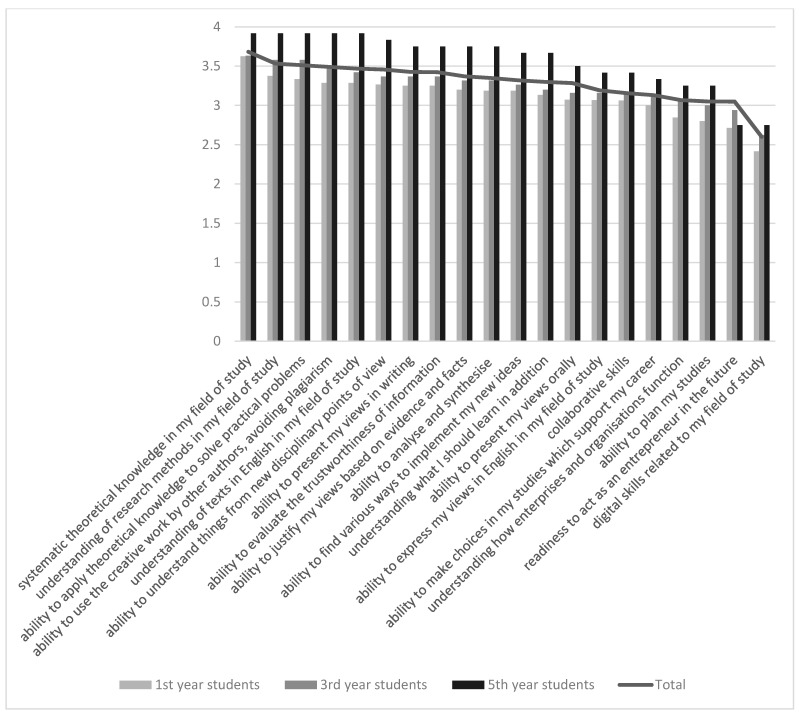
Development of competencies over the studies, average results: total, 1st, 3rd, and 5th year pharmacy students.

From the transferable skills, cooperation skills were named more often. Students were able to express their views to some extent more in writing than orally. Surprisingly, self-esteem in professional digital skills was lowest among all students in comparison to other competencies. Students also lacked knowledge in economics and business administration. The latter is also understandable as the compulsory professional entrepreneurship course was added to the Pharmacy curriculum during its update. To a small extent, the assessments of fifth year students were higher than other students that indicated the acquisition and improvement of both professional knowledge and transferable skills during their studies (Figure 4).

### 3.5. Support System

**Figure 5:**
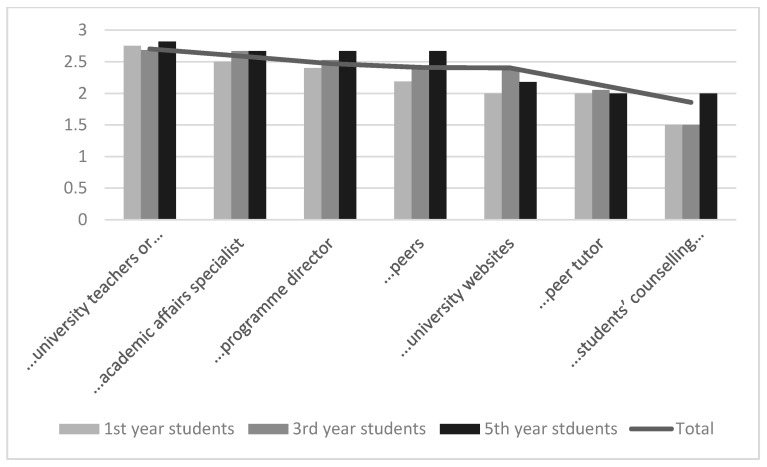
Use of support systems, average results: total, 1st, 3rd, and 5th year pharmacy students.

The use of support systems was not very common among students. Communication with fellow students was the highest in all academic years and the help of the UT counselling center was used the least (Figure 5).

### 3.6. Comparison of the Students’ Feedback at the Curriculum, Faculty and University Level

**Figure A1:**
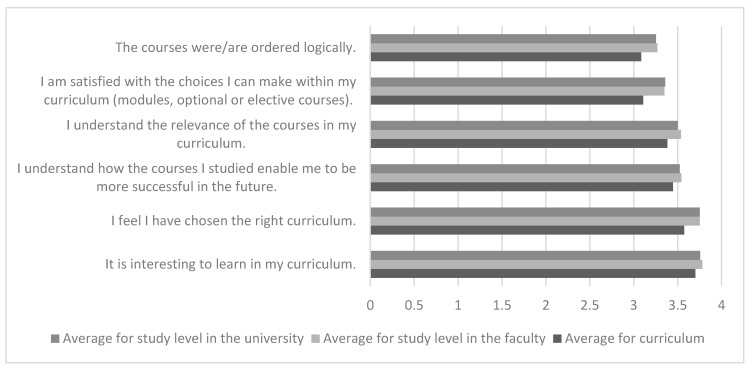
Ratings for the curricula, average results for the Pharmacy curriculum, in the Faculty of Medicine and at the University of Tartu (UT).

The feedback on curricula was higher for all statements at the faculty and university level than in the Pharmacy curriculum level. To some extent, pharmacy students were less satisfied with the selection and location of subjects and study modules in the curriculum than other students in the Faculty of Medicine and at the UT (Figure A1). 

**Figure A2:**
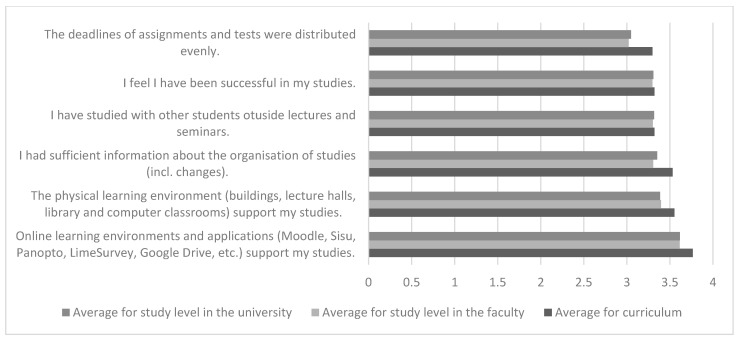
Ratings for the learning environment and academic affairs, average results for the Pharmacy curriculum, in the Faculty of Medicine and at the UT.

Pharmacy students rated both, the physical and online learning environment higher than other students from the UT. They were also more satisfied with information about organization of the studies and evenly distributed deadlines of assignments and tests (Figure A2). 

**Figure A3:**
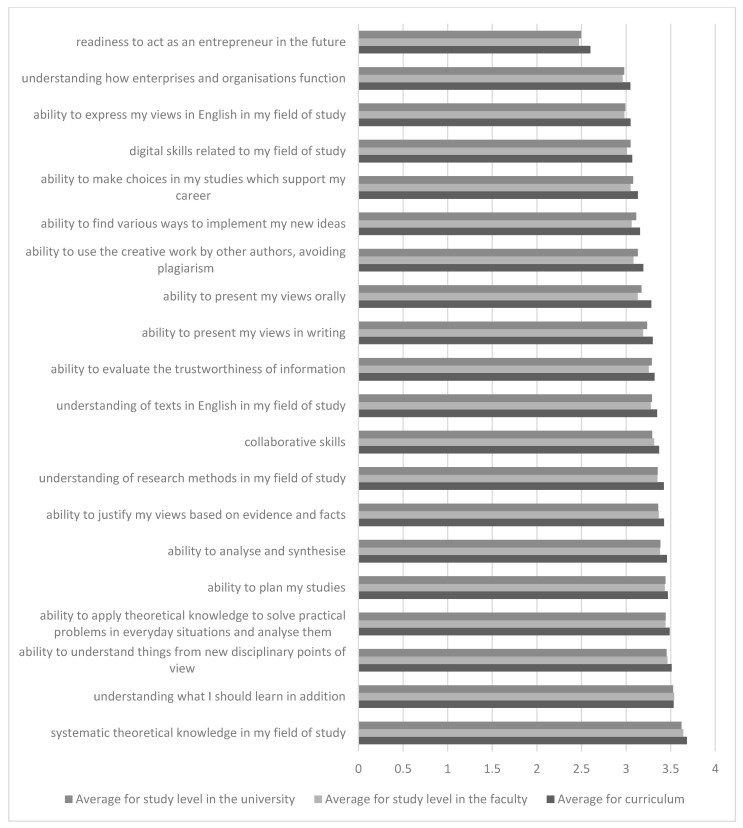
Development of competencies over the studies, average results for the Pharmacy curriculum, in the Faculty of Medicine and at the UT.

Development of competencies over the studies was similarly rated by all students participating in the survey. Interestingly, all responded students evaluated their understanding about entrepreneurship and organization functions rather high, but much less were willing to be self-employed entrepreneurs in the future (Figure A3).

**Figure A4:**
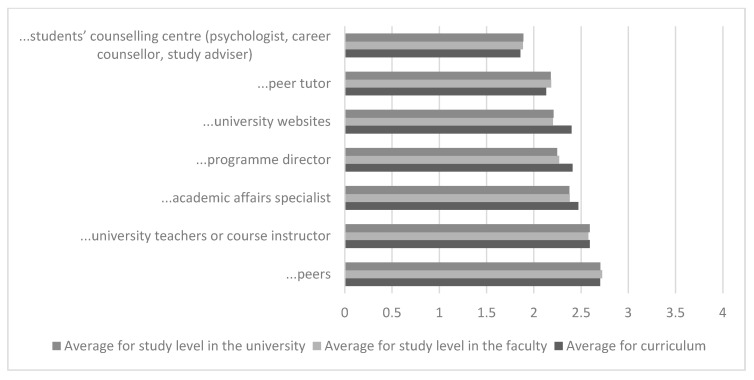
Use of support systems by students, average results for the Pharmacy curriculum, in the Faculty of Medicine and at the UT.

Similarly, to pharmacy students, other survey participants used support services rather infrequently and preferred communication with fellow students (Figure A4).

## 4. Discussion

Given the growing interest in CBPE, several studies have been performed worldwide to estimate whether student competencies are equally advanced in all required areas. In previous studies among pharmacy students in Europe, a comparison of first and fifth year students demonstrated more awareness of patient care competences within the final year students [20]. It was a first time to collect students’ feedback on the content and organization of all curricula at the UT. The study included first year students, whose opinions present rather expectations about the specialty being studied; third year students use knowledge and skills already gained, and fifth year students are in the best position to provide feedback on the acquired professional knowledge and the development of transferable skills. Most of the statements in this survey were mostly agreed by students. This shows that the curricula of the UT are well developed by content and structure. 

Within the framework of the curriculum survey conducted at the UT, it was possible to receive first views of pharmacy students on the CBPE in Estonia. Only first year students were able to give feedback on the updated Pharmacy curriculum, but nevertheless, it was possible to assess some of the differences between the previous and newly developed Pharmacy curriculum already. Implementation of CBPE should be continuously and regularly evaluated. For this purpose, several existing frameworks, as the European Pharmacy Competence Framework [14,15] and the International Pharmaceutical Federation (FIP) Global Competency Framework (GbCF) [21,22], could be seen as potential tools to use in the future. 

The results of this study did not clearly show the impact of the COVID-19 pandemic on the acquisition of professional and transferable skills. The results of another study at the UT showed that students’ learning outcomes did not change significantly, but there was a need to change the way subjects are assessed. The first outbreak of COVID-19 in spring 2020 has clearly had an impact on the conduct and organization of the internship. The number of partially and fully online curricula has increased in spring 2020 at the UT [23]. 

### 4.1. Curriculum

Despite of introducing pharmacy profession related subjects on the first year in the updated Pharmacy curriculum, the hesitant and slightly cautious attitude of first year students could be seen from the responses towards the selection of an interest about studying pharmacy. Pharmacy profession is still not always acknowledged as an integral part of healthcare in Estonia and the student candidates often miss an explicit understanding of the content and future career opportunities in the field of pharmacy. In order to increase the awareness of pharmacy education at the UT, the wording of the aims and outcomes of Pharmacy curriculum was updated, and the subjects were grouped into competence-based modules. The modules have been compiled based on the principles of CBPE curriculum design—longitudinal development of knowledge and skills [6]. One module contains subjects of different study years enabling to follow the development of particular competencies. Similar competence-based modules have been successfully introduced at the University of Helsinki in Finland [24]. There are several other examples of competency frameworks that have been used to evaluate knowledge and skills of both, entry level and practicing pharmacists. For example, in Croatia GbCF was used to develop national pharmacy competency framework. In Australia, competency framework for entry level pharmacists was compiled already in 1994. The general purpose of this document is to describe the roles and activities that are covered within the scope of practice for the pharmacy profession. Furthermore, it is used for the designing of pharmacy curricula. Such a broader approach, following the competency-based approach is much more valuable and has greater impact to the patient care and practice development in every level of pharmacy practice [25,26]. 

If to follow another principle of CBPE about integration of knowledge and skills, it is currently achieved more in later years of the Pharmacy curriculum (fourth and fifth study year). In this study, the higher satisfaction with the Pharmacy curriculum from the fifth year students confirms the previous considerations. 

### 4.2. Learning Environment

Compared to the respondents at the Faculty of Medicine and the UT level, pharmacy students were more satisfied with both the physical and electronic learning environment. For first year students, e-learning might be more difficult due to their need to adapt to new environments and arrangements, therefore, increased amount of independent learning may not be suitable for everyone during this period. On the other hand, students have recent experience in using various electronic devices, communication tools etc., from gymnasium. Digital skills and competences play essential roles in Estonian high school education and digital solutions support learning and teaching [27]. On the fifth year, students are obliged to use more self-directed learning as they need to complete research project, a six-months pharmacy internship, and take the final exam. More learning activities could be planned outside of university to encourage the use of professional knowledge in practical situations to support the principles of CBPE. It could be jointly done with flipped classroom model that is currently implemented successfully on a few courses at the Pharmacy curriculum [13].

### 4.3. Development of Competencies

While average ranking on competencies developed over studies on the Pharmacy curriculum, Faculty of Medicine and the UT level did not differ, among pharmacy students, fifth year students were to some extent more satisfied with acquired knowledge and skills than other respondents. To compare feedback about professional knowledge and transferrable skills, students reported more acquisition of theoretical knowledge and less practical and transferrable skills. For example, the rating about digital skills related to the field of studies was surprisingly low for all pharmacy students. The reasons of this result need further investigation. 

The transfer of theoretical knowledge to practice, reported more by fifth year students in this study, should be a pervasive principle in all study years of the Pharmacy curriculum. It helps students from the first year onwards to link the subjects to their future specialization and make important career choices at an early stage. 

Competencies about analytical skills, planning and the ability to assess information create a good foundation for critical thinking and evidence-based decision-making in professional life. These skills that were less reported by first and third year students, are important for the manager and the competent person in pharmaceutical industry, wholesale and retail sale of medicines. Collaboration skills, understanding of organizational mechanism, the ability to use the work of other authors and find ways to implement different ideas, oral and written communication skills contribute to an interdisciplinary work in all fields of pharmacy. Thus, the competencies that should be developed more in the future for pharmacy students are management (organization, research, etc.), leadership and innovation. Rapid technology changes, innovation, political and economic trends in the pharmacy field of Estonia require fast adaptation, forward thinking and exceptional leadership skills. Therefore, elective courses by other faculties at the UT, other universities in Estonia or internationally (e.g., digital pharmaceutical care) definitely help to enhance student leadership competencies and managerial skills. It is not only important to acquire professional competencies during undergraduate studies but to follow life-long learning (LLL) principles to advance professional development. In addition to CBPE, it is essential to develop practice based competency standards to support continuous professional development activities and thereof improve the quality of daily practice [28,29]. During pharmacy education and training, the principles of LLL are followed in general, but structured LLL system for pharmacists is under development. 

### 4.4. Support Systems

It is not common to use different possibilities of supporting system among students at the UT. As the counselling center was opened recently, the other alternatives (e.g., communication with university teachers and academic affairs specialists) have been available for decades. It is necessary to introduce the various support system possibilities to students to solve multiple questions for newcomers and reduce potential drop out. 

The UT has recently developed Good Practice of Learning and Good Practice of Teaching that could be used by all students and university teachers to develop scientific way of thinking and cooperation skills, creativity, entrepreneurship and self-analysis, and link theoretical learning to real life [30,31]. These principles support well CBPE. 

## 5. Conclusions

Students’ feedback to the Pharmacy curriculum at the UT provided important insight into the development of CBPE in Estonia. In the traditional course-based structure of the curriculum the principles of competency-based teaching and learning have been introduced through the implementation of competence modules supporting understanding about integration and longitudinal development of knowledge and skills. Several other changes support the application of theoretical knowledge in practice. In the future, more attention must be paid to the development of transferable skills, including digital skills. Continuous development of CBPE provide the basis for quality professional performance of the future pharmacists. 

## Data Availability

The data presented in this study are available on request from the corresponding author. The data are not publicly available due to privacy reason.

## Appendix A

**Table A1:** Ratings for the Pharmacy curriculum; to learning environment and academic affairs; development of competencies over the studies and use of support system, average results with SD: 1st, 3rd, and 5th year pharmacy students.

Category	1st Year Students		3rd Year Students		5th Year Students	
**Raitings for the pharmacy curriculum**	Average	SD	Average	SD	Average	SD
I feel I have chosen the right curriculum.	3.56	0.63	3.74	0.45	3.83	0.39
I understand how the courses I studied enable me to be more successful in the future.	3.5	0.52	3.63	0.63	3.83	0.45
It is interesting to learn in my curriculum.	3.38	0.79	3.21	0.49	3.83	0.39
I understand the relevance of the courses in my curriculum.	3.31	0.6	3.11	0.66	3.75	0.39
I am satisfied with the choices I can make within my curriculum (modules, optional or elective courses).	3.31	0.7	3	0.92	3.5	0.67
The courses were/are ordered logically.	3.19	0.75	2.79	0.92	2.92	0.67
**Raiting to learning environment and academic affairs**						
Online learning environments and applications (Moodle, Sisu, Panopto, LimeSurvey, Google Drive, etc.) support my studies.	3.63	0.5	3.79	0.42	3.92	0.29
The physical learning environment (buildings, lecture halls, library and computer classrooms) support my studies.	3.63	0.5	3.58	0.75	3.75	0.45
I had sufficient information about the organisation of studies (incl. changes).	3.5	0.52	3.32	0.51	3.58	0.67
I feel I have been successful in my studies.	3.31	0.54	3.32	0.56	3.58	0.51
The deadlines of assignments and tests were distributed evenly.	3.25	0.68	3.32	0.67	3.33	0.65
I have studied with other students otuside lectures and seminars.	3.19	0.7	3.26	0.95	3.33	1.07
**Raitings to development of competencies over the studies**						
systematic theoretical knowledge in my field of study	3.63	0.5	3.63	0.51	3.92	0.29
understanding of research methods in my field of study	3.38	0.54	3.58	0.51	3.92	0.29
ability to apply theoretical knowledge to solve practical problems in everyday situations and analyse them	3.33	0.45	3.58	0.76	3.92	0.29
ability to use the creative work by other authors, avoiding plagiarism	3.29	0.73	3.473684	0.76	3.92	0.29
understanding of texts in English in my field of study	3.29	0.61	3.42	0.5	3.92	0.29
ability to understand things from new disciplinary points of view	3.27	0.49	3.37	0.48	3.83	0.39
ability to present my views in writing	3.25	0.59	3.37	0.50	3.75	0.45
ability to evaluate the trustworthiness of information	3.25	0.59	3.37	0.60	3.75	0.45
**Raitings to development of competencies over the studies**						
ability to justify my views based on evidence and facts	3.2	0.56	3.32	0.56	3.75	0.45
ability to analyse and synthesise (distinguishing relevant aspects from irrelevant ones; creating connections; making inferences)	3.19	0.54	3.32	0.51	3.75	0.45
ability to find various ways to implement my new ideas	3.19	0.57	3.26	0.67	3.67	0.49
understanding what I should learn in addition	3.13	0.58	3.2	0.5	3.67	0.49
ability to present my views orally	3.07	0.67	3.16	0.60	3.5	0.52
ability to express my views in English in my field of study	3.07	0.9	3.16	0.83	3.42	0.51
ability to understand things from new disciplinary points of view	3.27	0.49	3.37	0.48	3.83	0.39
ability to present my views in writing	3.25	0.59	3.368	0.50	3.75	0.45
ability to evaluate the trustworthiness of information	3.25	0.59	3.37	0.60	3.75	0.45
ability to present my views in writing	3.25	0.59	3.37	0.50	3.75	0.45
ability to evaluate the trustworthiness of information	3.25	0.59	3.37	0.60	3.75	0.45
collaborative skills	3.06	0.81	3.16	0.61	3.42	0.51
ability to make choices in my studies which support my career	3	0.74	3.11	0.42	3.33	0.78
understanding how enterprises and organisations function	2.85	0.73	3.06	0.56	3.25	0.75
ability to plan my studies	2.8	0.65	3	0.61	3.25	0.87
readiness to act as an entrepreneur in the future	2.71	0.67	2.94	0.72	2.75	0.75
digital skills related to my field of study	2.42	0.76	2.63	0.67	2.75	0.75
**Raitings to use of support system**						
university teachers or course instructor	2.75	0.7	2.68	0.51	2.82	0.65
academic affairs specialist	2.5	0.54	2.67	1.3	2.67	1.36
programme director	2.4	1.09	2.53	1.34	2.67	1.44
peers	2.19	0.45	2.44	0.48	2.67	0.49
university websites	2	0.40	2.36	0.41	2.18	0.74
peer tutor	2	1.32	2.05	1.3	2	0.58
students’ counselling centre (psychologist, career counsellor, study adviser)	1.5	0.68	1.5	0.5	2	1

## Appendix B

Student feedback to curricula content and structure on the level of Pharmacy curriculum, Faculty of Medicine and University of Tartu
